# Survey of Phenolic Acids, Flavonoids and In Vitro Antioxidant Potency Between Fig Peels and Pulps: Chemical and Chemometric Approach

**DOI:** 10.3390/molecules26092574

**Published:** 2021-04-28

**Authors:** Lahcen Hssaini, Francisca Hernandez, Manuel Viuda-Martos, Jamal Charafi, Rachid Razouk, Karim Houmanat, Rachida Ouaabou, Said Ennahli, Driss Elothmani, Ilham Hmid, Marie Laure Fauconnier, Hafida Hanine

**Affiliations:** 1 National Institute for Agricultural Research (INRA), Meknes BO 578, Morocco; jcharafi@gmail.com (J.C.); razouk01@yahoo.fr (R.R.); k.houmanat@gmail.com (K.H.); 2 Laboratory of Bioprocess and Bio-Interfaces, Faculty of Science and Technics, Beni-Mellal BO 523, Morocco; hmid.ilham@gmail.com; 3 Grupo de Investigación de Producción Vegetal y Tecnología, Dpto. Producción Vegetal y Microbiología, Ecuela Politécnica Superior de Orihuela, Universidad Miguel Hernández de Elche, Ctra. de Beniel, km 3,2, E-03312 Orihuela, Alicante, Spain; francisca.hernandez@umh.es; 4 Dpto. Tecnología Agroalimentaria, IPOA. Escuela Politécnica Superior de Orihuela, Universidad Miguel Hernández de Elche, Ctra. de Beniel, km 3,2, E-03312 Orihuela, Alicante, Spain; mviuda@umh.es; 5 Department of Chemistry, Faculty of Science Semlalia, Cadi Ayyad University, Marrakesh BO 2390, Morocco; rachaouaabou@gmail.com; 6 National School of Agriculture (ENA), Meknes BO S/40, Morocco; ennahlisaid@gmail.com; 7 USC 1422 GRAPPE, INRAE, Ecole Supérieure d’Agricultures, SFR 4207, QUASAV, 55 rue Rabelais, 49100 Angers, France; d.elothmani@groupe-esa.com; 8 Laboratory of Volatolomic, Department of General and Organic Chemistry Passage des Déportés 2, 5030 Gembloux, Belgium; marie-laure.fauconnier@ulg.ac.be

**Keywords:** phenolic acids, flavonoids, antioxidant activity, *Ficus carica* L., chemometric, heatmap

## Abstract

In the present study, chromatic coordinates, phenolic acids, flavonoids and antioxidant capacity assessed by 1,1-diphenyl-2-picrylhydrazyl (DPPH), 2,2′-azino-bis (3-ethylbenzothiazoline-6-sulfonate (ABTS) and lipid peroxidation inhibition capacity (LPIC) essays and their relative IC50 were investigated in 25 fig cultivars growing in Morocco. The aims of this study were to determine (i) the variation in these compounds among light and dark-colored cultivars, (ii) their partitioning between fruit peel and pulp and (iii) to display network connections among these variables. Twelve phenolic compounds (PCs) were isolated in peel extract versus eight in pulp samples. Anthocyanins, mainly cyanidin-3,5-diglucoside and cyanidin-3-*O*-rutinoside, were the predominant compounds in peels, where the mean concentrations were 75.90 ± 18.76 and 77.97 ± 18.95 µg/g dw, respectively. On the other hand, (−)-epicatechin and cyanidin-3-*O*-rutinoside were the major compounds in the pulp extracts, where the mean values were 5.23 ± 4.03 and 9.01 ± 5.67 µg/g dw, respectively. A two-dimensional hierarchically clustered heatmap was applied to the dataset to explore correlations in the dataset and similarities between cultivars, without dimensionality reduction. Results showed that anthocyanins, particularly pelargonidin-3-*O*-rutinoside, cyanidin-3,5-diglucoside and cyanidin-3-*O*-rutinoside, were the main contributors to the peels’ free radical scavenging capacity. This capacity was particularly higher in the peel of dark-colored figs compared to the fruit pulp. The local cultivar “INRA 1301” showed the most promising phenolic profile due to its very high levels of almost all detected PCs, especially (−)-epicatechin, quercetin-3-*O*-rutinoside, quercetin-3-*O*-glucoside, cyanidine-3,5-diglucoside, cyanidine-3-*O*-rutinoside and pelargonidin-3-*O*-rutinoside (54.66, 141.08, 35.48, 494.08, 478.66, 12.56 µg/g dw, respectively). Having the darkest figs in the collection (L* = 25.72, c* = 22.09 and h° = 20.99), this cultivar has also combined promising IC50 values, which were of 19.85, 40.58 and 124.78 µg/mL for DPPH, ABTS and LPIC essays, respectively.

## 1. Introduction

The ever-growing interest in functional foods, particularly underutilized fruits, is based on their uniqueness of the natural biological resources necessary to enhance human health and well-being. Worldwide, large species are not fully assessed for their nutritional values and biologically active compounds involved in the consumer health promotion, so far. Although the naturally occurring phenotypic, chemotypic and ecotypic diversity of most of these species is still scarcely studied, it is evident that they present an invaluable potential source of bioactive compounds directly associated to the prevention of coronary diseases. Particular attention should be devoted to the investigation of secondary metabolites of these species, since they not only present the main quality indicators of new cultivars but are also important in chemotaxonomy [1]. One of the major secondary metabolites are phenolic compounds that belong to the large group of phytochemicals widespread in plants and plant derived foods and beverages [2]. They have a large structural and functional diversity and can be classified into water-soluble compounds (phenolic acids, phenylpropanoids, flavonoids and quinones) and water-insoluble compounds (condensed tannins, lignins and hydroxycinammic acids) [3]. They represent the second most abundant group of organic compounds in the plant genera [4]. Some of them are extremely widespread, while others are found in certain plant families or organs or at specific ripening stages [4]. These molecules have a critical role in plant defense mechanisms such as biotech stress, particularly pathogen or insect attack (i.e., proanthocyanidins, condensed and hydrolysable tannins), and ultraviolet irradiation (i.e., flavonols) [5,6,7]. They are also associated to the sensory, color, flavor and astringency of foods. Anthocyanins in particular are responsible for the colors of various plant parts such as flowers, leaves and especially fruits with blue, purple or red peels [8]. The increasing interest in these compounds is mainly correlated to their antioxidant potential and their specific role in the prevention of some diseases due to their multiple biological effects, such as scavenging the free radicals from cell metabolism, antimutagenic and/or anticarcinogenic activities and anti-inflammatory action [9]. Even at low concentrations, phenol compounds may be great contributors to human health [4]. Recent studies have stressed the importance of diet rich in phenolic compounds in prevention of the oxidative stress and metabolic diseases such as atherosclerosis, cancer and chronic inflammation [10,11,12,13,14]. Phenolic compounds concentration, distribution and their antioxidant potential are strongly dependent on the cultivar, degree of ripeness, geographic location, horticultural practices and pre and postharvest conditions [15].

According to the United States Department of Agriculture database (USDA), figs (*Ficus carica* L.), emblematic food in the Mediterranean diet [16], are among fruits that presents the highest values of phenolic compounds [17]. Since they are among various agroecosystems, figs are one of the major natural sources of bioactive compounds in the health-promoting Mediterranean diet for millennia [18]. Red wine and tea, which are well-publicized polyphenol sources, comprise lower concentrations than figs [19]. Anthocyanin content, mainly cyanidin-3-rutinoside; flavanols, particularly quercetin-rutinoside; phenolic acids such as chlorogenic acid; flavones such as luteolin and apigenin-rutinoside are reported as the main phenolic compounds identified in fresh figs [20]. Morocco, the third fig producer with more than 85,172 tons, is identified in historical sources as a fig cultivation area and is still today one of the most important fig diversity hotspots, which contains a large number of typical local varieties [21]. This germplasm was basically assessed using morphological and molecular markers. However, phenolic compounds assessment and in vitro antioxidant activity investigation using several tests are still lacking. Furthermore, to the best of our knowledge, there are very few reports investigating the repartitioning of these proprieties among fig peels and pulps and their correlation to the external and internal chromatic attributes. Therefore, the main aims of the present study were to (i) determine the phenolic compounds and in vitro antioxidant activity over peels and pulps of an ex-situ fig collection of 25 cultivars, (ii) investigate their concentration and availability between the two parts of the fruit, (iii) determine potential correlations with antioxidant potency and chromatic coordinates and finally (iv) to identify the most discriminant of them. Despite numerous studies reported the phenolic profile of fig accessions growing worldwide [17,18,19,20,21], very few reports have addressed the bioavailability of these bioactive compounds with respect to their partitioning in different parts of the fruit, within a large screening scheme aiming to correlate them to antioxidant potency with regards to their chromatic coordinates and the phenotypic factor. In this respect, this work is the first report on fig chemotypic diversity based on the partitioning of phenolic compounds, chromatic coordinates and antioxidant activity between peel and pulp of a large sample number of cultivars growing in Morocco, using a chemometric approach.

## 2. Results and Discussion

### 2.1. Peels and Pulps Color

**Figure 1:**
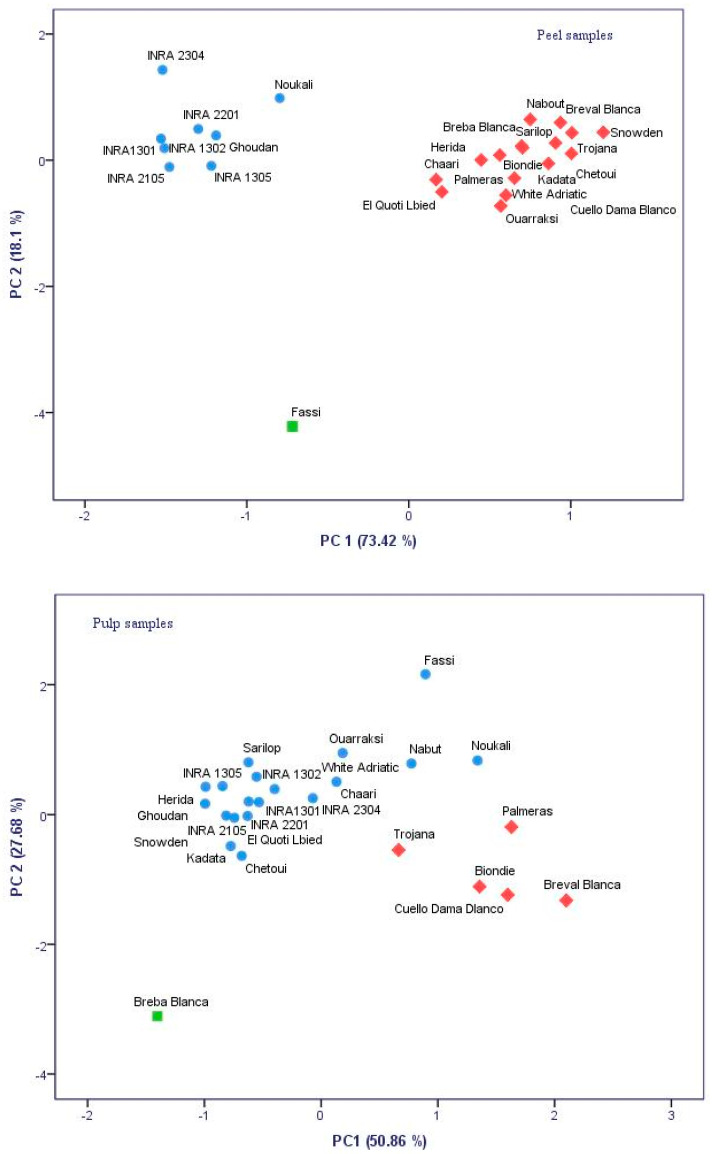
Principal component analysis (PCA) two-dimensional scatter plots based on the first two principal components (PC1 and PC2) generated for 25 cultivars based chromatic coordinates color of figs’ peels and pulps.

The fig peels and pulps color showed significant differences among cultivars at *p* < 0.001, with the exception of the pulp lightness coordinate (L*) (Table 1). Therefore, peels’ chromatic coordinates present more accurate discrimination between cultivars than pulp color coordinates. Overall, peel color varied from bright yellow color (high and positive values of L* and c*) to atypical dark and blue purple color (negative L* and c* and high values of the hue). While in pulp, the color varied from pale pink (high values of L*, positive values of a* and b*) to dark red (low L* and c* and high positive a*). The cultivar “Trojana” had the brightest peels with the coordinates L* and c* recorded the highest values (73.15 and 50.94, respectively), whereas “INRA 1301” had the darkest colored figs (L* = 25.72 and c* = 22.09). Regarding pulp samples, the cultivars “Fassi” and “Breval Blanca” had the darkest color, where L* recorded the lowest values (18.6 and 19.05, respectively). All cultivars were classified based on their fruits’ peels and pulps characterization using principal component analysis (Figure 1). Inspection of scatterplots showed that peels color displayed outlying subsets more than the pulp. Hence, the total variance obtained with peels data was of 91.51%, while pulps characterization accounted about 78.54%. In fact, the principal component analysis (PCA) scatterplot for peels’ chromatic coordinates splits the samples into two main groups describing blue-purple and light-colored cultivars. Having the lowest chroma value, the local cultivar “Fassi” was largely distinguished from the other subsets. However, a pulp samples scatterplot showed low discrimination resolution. Therefore, peel and pulp color evaluation using these coordinates is of great importance in fruits quality assessment. Several studies highlighted the importance of these descriptors to explore potential correlations between them and some antioxidant compounds, mainly phenols (anthocanins, tanins, catechins, etc.) and carotenoids (lycopene, beta-carotene, etc.) [22,23].

### 2.2. Spectrophotometric Assays

Total phenols (TPC), total flavonoids (TFC), total anthocyanins (TAC) and total proanthcyanidins content (TPAC) showed highly significant differences among cultivars, depending on their fruit parts (*p* < 0.001) (Table 1). These compounds were more than two times higher in fruits peels compared to their pulps, as observed in other fruits such as quince [24] and apricot [25]. This may suggest that peels are responsible of the higher level of figs total phenolics. A wide range of concentrations were obtained in both fruit parts except for TPAC, which showed a narrowed concentration interval (Table 1).

In peels, TPC varied between 370 and 3162.86 mg GAE/100 g dw, while TFC were in the range of 188.57 and 2013.57 mg CE/100 g dw. TAC was highly abundant in dark samples and ranged between 4.14 and 192.5 mg cyanidin-3-rutinoside/100 g dw. In pulps extracts, TPC, TFC and TAC were in the range of 105.71–1255.71 mg GAE/100 g dw, 13.57–331.43 mg CE/100 g dw and 2.27–19.44 mg cyanidin/100 g dw, respectively. For both fruit parts, TPAC varied within a narrow interval of 0.2–3.09 and 0.2–1.06 mg cyanidin/100 g dw. Generally, they were present in high amounts in purple pulps when compared to light-colored ones. It is noteworthy that proanthocyanidins are quantified in all pulps samples as the same as the peels, which is probably due to the fact that they are the key determinant for red color in pulp and purple and blue colors skin fruits as well as anthocyanins [26].

The local cultivar “INRA 1301” combined the highest levels of TPC, TFC, TAC and TPCA in its peels, where the mean values were of 2860.48 mg GAE/100 g dw, 1944.52 mg CE/100 g dw, 192.23 mg cyanidin-3-rutinoside/100 g dw and 2.59 mg cyanidin/100 g dw, respectively (Table 2; Table 3). Regarding the pulps, the local cultivar “Ghoudan” combined the highest amounts of TPC and TFC, where the mean concentrations were of 1186.67 mg GAE/100 g dw and 271.90 mg CE/100 g dw (Table 2 and Table 3). It is noteworthy that these compounds were found to be more abundant in dark-colored peels compared to light-colored ones, which is not always in the same sense regarding the fig pulps.

These results are consistent with those of Çalişkan and Polat. [27], who reported that purple and black figs hold higher phenolic amounts than the green and yellow ones. The same observation was reported with Italian figs by Del Caro and Piga [28] and Turkish ones where dark-colored fruits were mentioned to have higher levels of total phenols, flavonoids and anthocyanins than the light-colored ones, and those amounts were mainly concentrated in the peels [29]. The significant difference between cultivars and their fruits peels’ and pulps’ phenolics contents has also been previously found by Harzallah et al. [30] in three fig varieties growing in Tunisia and by Palmeira et al. [31] in the Portuguese variety “Pingo de Mel”. These authors reported that the amounts of phytochemicals compounds are usually dependent not only on the variety but also differ significantly from one fruit part to the other. According to the same authors, the fig antioxidant potency seemed also to be mainly related to the peel part compared to the pulp part. The same result was reported in other consumed fruits, such as apricots [25], quinces [24], nectarines, plums and peaches [32] and was mainly related to the genetic factor.

In the industrial processing of figs, the pulp is used, whilst the peel is usually discarded [33], which generates a significant volume of byproducts consisting mainly of peels. In the studies conducted by Viuda-Martos et al. [34] and Buenrostro-Figueroa et al. [31], it was proven that these byproducts have abundant phytochemical compounds, which suggests their valorization and exploitation as nutraceuticals.

### 2.3. In Vitro Antioxidant Activity

Results of the free-radical-scavenging effect of figs’ peel and pulp extracts on DPPH• and ABTS•+ radicals and lipid peroxidation inhibition are summarized in Table 1, Table 2 and Table 3. They are expressed as Trolox equivalent per g of dry weight and by the antioxidant concentration required for a 50% of radical reduction (IC50), so that a lower value of IC50 indicated a higher antioxidant activity and vice versa. These methods were combined to obtain an overview of figs antioxidant capacity, since no single assay can fully characterize the profile of each sample [9]. Both the peel and pulp samples were proven to have antioxidant activities with significant differences (*p* < 0.001) among all cultivars (Table 1). In the DPPH assay, the values ranged from 21.23 to 367.26 mMol TE/g dw for peel samples, which is at least two times higher than the scavenging capacity exhibited by pulp samples, where the average concentrations ranged between 13.92 and 151.24 mMol TE/g dw (Table 2). Regarding peel samples, the variety “Cuello Dama Blanca” recorded the highest antioxidant activity (AA) followed by “Fassi”, where the average values were of 333.99 and 332.13 mMol TE/g dw, respectively (Table 3). Whereas “Trojana” and “INRA 2304′ exhibited the lowest AA (5.27 and 16.62 mMol TE/g dw, respectively). The pulp extracts present the low DPPH• scavenging activity, where “White Adriati” and “Chetoui” showed the highest values (121.65 and 104.73 mMol TE/g dw, respectively) (Table 3).

The ABTS assay showed a wide range of variation for both peel and pulp antiradical capacity (7.57–563.53 and 6.59–207.49 mMol TE/g dw, respectively) (Table 2). Peels of the cultivars “Chaari” and “Fassi” showed the highest AA (527.25 and 493.69 mMol TE/g, respectively), while “Cuello Dama Blanca” and “Snowden” fig pulps exhibited the highest AA, where the values were 204.68 and 160.43 mMol TE/g, respectively (Table 2 and Table 3).

The lipid peroxidation inhibitory effects of both fig parts were significantly different among cultivars and, generally, showed a narrow interval of variation compared to the other assays. Hence, in peels, the lipid peroxidation inhibition capacity (LPIC) was in the range of 139.17 and 353.11 mMol TE/g dw, whereas in pulps, it ranged between 42.89 and 226.88 mMol TE/g dw, respectively (Table 2). Peels of “Kadota” and “Ghoudan” exhibited the highest LPIC (301.76 and 289.63 mMol TE/g dw, respectively), while “Bioudie” and “White Adriatic” had the lowest values (154.84 and 156.27 mMol TE/g dw, respectively). Similarly, pulps extracts displayed low LPIC compared to the peels, where “INRA 1305” and “Bioudie” showed the highest values (189.08 and 147.81 mMol TE/g dw, respectively), while “Sarilop” and “Ournaksi” recorded the lowest ones (57.85 and 67.12 mMol TE/g dw, respectively) (Table 2 and Table 3). To conclude, among all assays, figs’ peels seem to be the main contributors to the antioxidant capacity comparing to their pulps. In addition, dark-colored peels exhibited the highest antioxidant capacity compared to the light-colored ones. These results were similar to those reported by Solomon et al. [29], Pande and Akoh, Ammar et al., Konak et al. [35,36,37], where several methodologies have been employed to assess the in vitro antioxidant capacity of different fig parts. It is noteworthy that such in vitro antioxidant assays are semi-quantitative and do not always represent the in vivo antioxidant capacity [38].

### 2.4. The Half Maximal Inhibitory Concentration (I50)

The IC50 is a variable that reflects the quality of radical scavenging for each of the antioxidant tests. The antioxidant potency, inversely proportional to the IC50 value, is more important when very small concentrations are required to scavenge half of the radicals [13]. The IC50 results for both peel and pulp samples are summarized in Table 1, Table 2 and Table 3. Indeed, significant divergences were spotted between sampled fruits following the cultivars and the fruit part investigated (*p* > 0.001) (Table 1). It is noteworthy that in all antioxidant assays, peels required very low concentrations to scavenge half of radicals, compared to pulp extracts. However, there are very few exceptions to this rule, where the pulps extracts had a higher IC50 values. In this case, “Breval Blanca”, “El Quoti Lbied” and “Kadota” exhibited higher DPPH IC50 values in their pulps compared to the peels’ extracts. Similarly, the local cultivars “Fassi” and “INRA 2201′ showed a higher LPIC IC50 in their pulps’ extracts than their peels. It should be noted that the first three cultivars have light-colored figs, whereas the last two give dark-colored fruits (Table 2; Table 3). A similar result was found by Harzallah et al. [30], who reported that in DPPH assay, the IC50 purple pulps of some fig varieties were a little higher than their peels. It is probable that these differences are due to the partitioning of the phenolic compounds between both fruit parts and the radical scavenging potency of each compound [39]. Among the 25 cultivars, the dark-colored peels of “INRA 2304” combined the lowest IC50 values for both DPPH (2.12 µg/mL) and ABTS (21.84 µg/mL) assays, which means that its peels required very low concentrations to scavenge 50% of free radicals. Taking all the assays together, the local cultivar “INRA 1302” peels combined the most promising IC 50 values, where the concentrations were of 3.97, 56.49 and 76.19 µg/mL, respectively, for DPPH, ABTS and LPIC assays (Table 2). However, no cultivar had a similar combination for the pulps’ extracts. It is noteworthy that among all antioxidant assays, DPPH test had the lowest values of IC50, while ABTS showed the highest ones (Table 2; Table 3).

Even consumers usually prefer fruits with attractive appearance, especially the peels’ color, they tend, while eating the fruit, to remove the peel; however, this fruit part is evidently the major source of phenolic compounds that highly contribute to the antioxidant capacity and systematically protect against diseases related to oxidative stress. The consumption of the whole figs is clearly an important habit for promoting the health promoting diet in Mediterranean society [30].

### 2.5. Polyphenolic Profile

**Figure 2:**
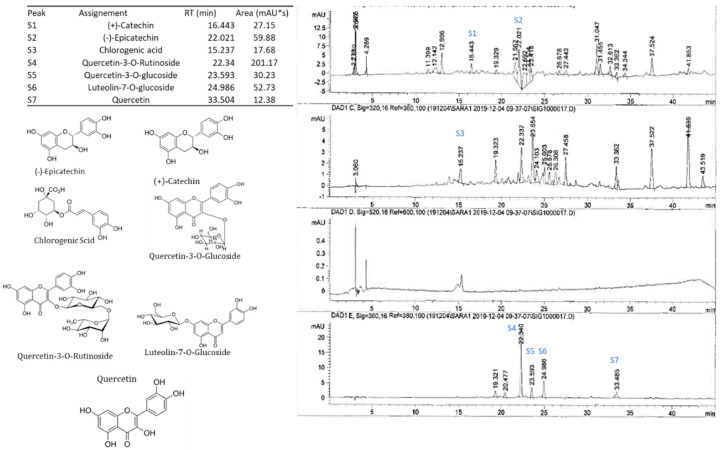
HPLC-DAD profile, chemical structures of the main phenolic compounds identified in the fig pulp. Example of the cultivar INRA 2015.

**Figure 3:**
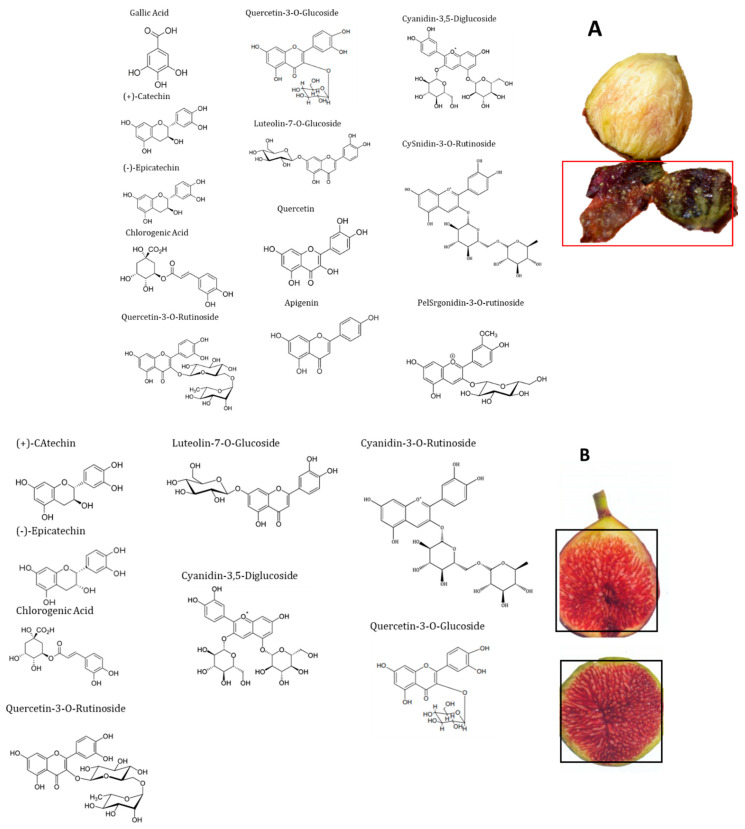
Chemical structures of the main phenolic compounds identified in the fig peels (**A**) and pulp (**B**).

High-performance liquid chromatography (HPLC) with a diode-array detector (DAD) analyses showed the presence of several phenolic compounds belonging to phenolic acids (hydroxycinnamic acid and hydroxybenzoic acid derivatives) and flavonoids (flavonols, flavones and anthocyanidins). Indeed, eight phenolic compounds, including: (+)-catechin, (−)-epicatechin, chlorogenic acid, quercetin-3-*O*-rutinoside, quercetin-3-*O*-glucoside, luteolin-7-*O*-glucoside, cyanidin-3,5-diglucoside and cyanidin-3-*O*-rutinoside, were detected in the pulp. While in peel extract, twelve compounds were isolated (gallic acid, (+)-catechin, (−)-epicatechin, chlorogenic acid, quercetin-3-*O*-rutinoside, quercetin-3-*O*-glucoside, luteolin-7-*O*-glucoside, quercetin, apigenin, cyanidin-3,5-diglucoside, cyanidin-3-*O*-rutinoside and pelargonidine-3-*O*-rutinoside) (Figure 2 and Figure 3). These compounds showed significant differences among cultivars and fruits parts (*p* < 0.001) (Table 1). These results were similar to those reported by Vallejo et al. [20], Viuda-Martos et al. [34] and Harzallah et al. [30].

Among all cultivars, the PCs’ concentrations were higher in peels compare to pulps extracts. Anthocyanins, particularly cyanidin-3,5-diglucoside and cyanidin-3-*O*-rutinoside, were the predominant compounds in peels, where the mean concentrations were 75.902 ± 18.76 and 77.972 ± 18.95 µg/g dw, respectively. For flavonols, only (−)-epicatechin, quercetin-3-*O*-rutinoside and quercetin-3-*O*-glucoside were detected. Gallic acid and pelargonidin-3-O-rutinoside were only detected in the local cultivars “Chetoui” and “Nabout”, with the respective levels of 8.363 ± 1.88 and 6.731 ± 2.019 µg/g dw (Table 4). These results agree with those reported for peels of the Portuguese variety “Pingo de Mel” by Palmeira et al. [31]. The local cultivar “INRA 1301” presented the most interesting phenolic profile due to its very high levels of almost all detected PCs, especially (−)-epicatechin, quercetin-3-*O*-rutinoside, quercetin-3-*O*-glucoside, cyanidine-3,5-diglucoside and cyanidine-3-*O*-rutinoside, with the main concentrations of 54.66, 141.08, 35.48, 494.08 and 478.66 µg/g dw, respectively (Table 4). Likewise, the Spanish variety “Cuello Dama Blanca” combined the highest levels of chlorogenic acid, luteolin-7-*O*-glucoside, quercetin and apigenin 8.76, 17.9, 59.52 and 4.84 µg/g dw, respectively.

In pulps extracts, (−)-epicatechin and cyanidin-3-*O*-rutinoside were the major compounds. They were detected in all cultivars at high levels (5.23 ± 4.03 and 9.01 ± 5.67 µg/g dw, respectively). Cyanidin-3,5-diglucoside were the third predominant compound, that ranged from 0.81 to 28.45 µg/g dw, with a mean of 6.06 ± 6.71 µg/g dw, followed by (+)-catechin and chlorogenic acid (1.93 ± 1.29 and 1.01 ± 1.16 µg/g dw, respectively). However, luteolin-7-*O*-glucoside was detected in only two cultivars, “Chetoui” and “Palmeras”, with respective concentrations of 0.75 ± 0.35 and 4.47 ± 0.04 µg/g dw (Table 5). These results are generally in agreement with those of Del Caro and Piga. [28], who used the same method on the Italian varieties “Mattalon” and “San Pietro”. These concentrations, mainly of (+)-Catechin, cyanidin-3-*O*-rutinoside and luteolin-7-O-glucoside, are higher in comparison with bananas, pears and apples; however, they are similar to black grapes [40].

In the study of Palmeira et al. [31], rutin (quercetin-3-*O*-rutinoside) was the predominant compound in fig skin, in contrast to our results, where cyanidine-3,5-diglucoside and cyanidine-3-*O*-rutinoside were predominant. Several works on the species have shown that PCs are strongly dependent on the cultivar but is also influenced by other factors including the fraction analyzed (pulp, peel or juice), the ripening stage and the growing conditions [15,16,30]. This is consistent with the results of Solomon et al. and Del Caro and Piga [28,29]. Finally, the PCs in fig pulp represent about 20% of the total concentration in the whole fruit. It is noteworthy that these are the first results reported regarding figs’ phenolic composition and their partitioning between the peel and the pulp of a large fig cultivar numbers growing under Moroccan climate, with respect to their antioxidant, chromatic coordinates. The herein reported findings are of great importance for efficient nutraceutical use of these raw materials.

### 2.6. Heat Map Analysis

Data visualization is an essential tool for biochemical data analysis, and dimensionality reduction methods, such as principal component analysis (PCA), are usually used to draw high dimensional data onto two- or three-dimensional space so it can be visualized. However, this transition is costly, often resulting in loss of the total variance. A hierarchically clustered heatmap is one of numerous analyses that does not need a dimensionality reduction to visualize data. It is a widely used technique to analyze complex biological data by displaying network connections in a symmetric adjacency matrix [41].

**Figure 4:**
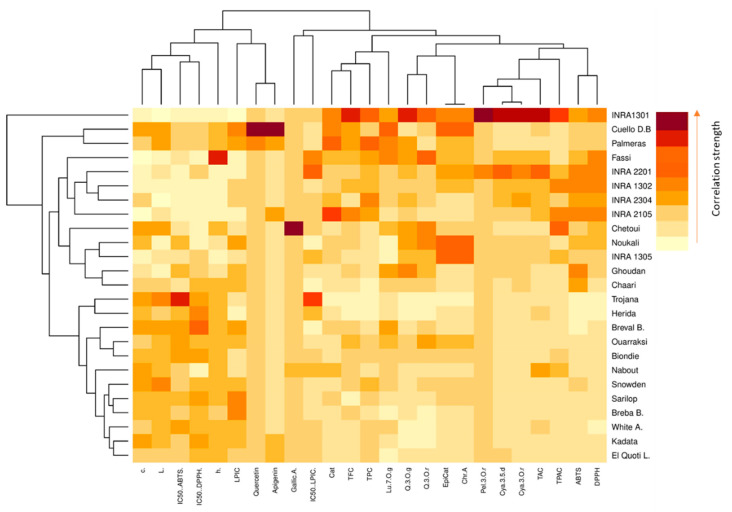

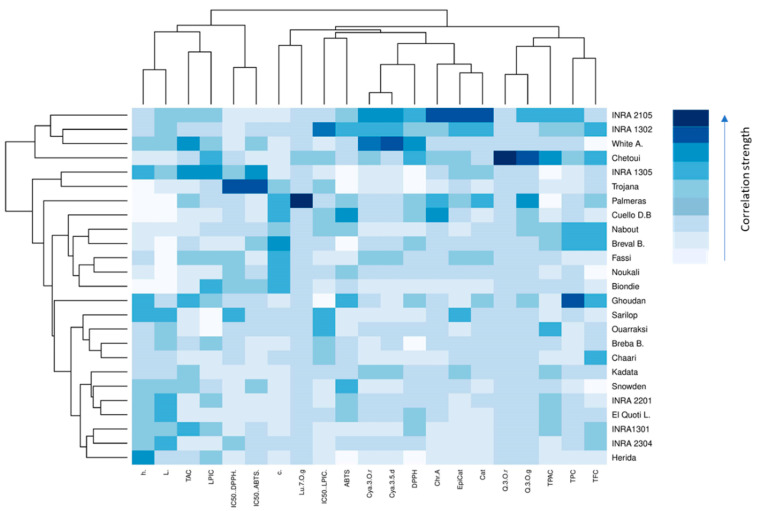
Hierarchically clustered heatmap based on the correlation matrix of studied variables in both peel (**red map**) and pulp (**blue map**). The low color intensity means the lower value and vice versa. Chr.A: chlorogenic acid; Q.3.O.r: quercetin-3-*O*-rutinoside; Q.3.O.g: quercetin-3-*O*-glucoside; Lu.7.O.g: luteolin-7-*O*-glucoside; Quercetin: quercetin; Apigenin: apigenin; Cya.3,5.d: cyanidin-3,5-diglucoside; Cya.3.O.r: cyanidin-3-*O*-rutinoside; Pel.3.O.r: pelargonidin-3-O-rutinoside. White A.: “White Adriatic”; Cuello B.D: “Cuello Dama Blanca”; Breval B.: “Breval Blanca”; Breba B.: “Breba Blanca”; El Quoti L.: “El Quoti Lbied”.

Color-coded two-dimensional heatmaps for both fruit parts are formed with two clusters using Euclidean distance following Ward method; one is sample-oriented while the other is variable-oriented (Figure 4). In this figure, weak correlations between studied variables are displayed in low color intensity, while stronger ones are shown with high color intensity. Cultivars and variables clustering as well as the correlations among dataset were quite different between fig peel and pulp. In pulp samples, the chromatic coordinates (L*, c*, h°) were clustered with LPIC and the IC50 of DPPH and ABTS assays, which are correlated to quercetin and apigenin. These compounds seem to have a large effect on the peel antioxidant potency (Figure S1). These variables tend to be higher in the cultivars “Trojana”, “Breval Blnaca”, “Ournaksi”, “Bioudie” and “Nabout” that constitute, among others, a distinctive cluster. On the other hand, catechin, luteolin-7-O-glucoside, quercetin-3-*O*-rutinoside, epicatechin and chlorogenic acid were clustered together and correlated to TPC, TFC and IC50 (LPIC). These compounds showed similar tendencies to be accumulated by the local cultivars “Chetoui”, “Noukali”, “INRA 2305′, “Ghoudan” and “Chaari”, which constitute a homogenous cluster. It is noteworthy that these cultivars are characterized by dark-colored figs, which are known to hold abundant amounts of these compounds. Pelargonidin-3-O-rutinoside, canidin-3,5-diglucoside and cyanidin-3-*O*-rutinoside are anthocyanins that were clustered together with total proanthocyanins and revealed a strong correlation to the free radical scavenging capacity of peel extracts (Figure S2). The pigments belong to the flavonoid class and seem to be the major contributors to the free radical scavenging process of fig peels. The local cultivar “INRA 1301” is clustered as a single branch and therefore largely distinguished from the other clusters. It combined the highest levels of flavonoids compounds and consequently showed high level of DPPH• and ABTS•+ radical scavenging capacity. This cultivar has dark-colored fruits, which is in accordance with several studies, which showed that fig skins have much higher amounts of phytochemical compounds, mainly flavonoids, which strongly contribute to the antioxidant capacity [18,29,30,35,37].

The pulp heatmap showed a different spatial distribution of individuals and variables, where catechin, epicatechin, chlorogenic acid, cyanidine-3.5-diglucoside and cyanidine-3-*O*-rutinoside were the highly correlated variables, which were related to the free radical scavenging capacity. The cluster composed of the cultivars “INRA 2105”, “INRA 1302” and “White Adriatic” showed similar tendencies to accumulate these variables. “Chetoui”, the nearest neighbor to this cluster, combined the highest level of quercetin-3-*O*-rutinoside, quercetin-3-*O*-glucoside and TPAC. The other cultivars were essentially clustered based on their pulp chromatic coordinates that seemed moderately correlated to TAC and the antioxidant potential.

## 3. Materials and Methods

### 3.1. Plant Material

Figs of an ex-situ collection were randomly harvested at their full maturity (August–September of 2018). The collection is composed of 16 local and 9 introduced varieties and was planted in complete randomized block in the experimental station on the National Institute for Agricultural Research of Meknes (INRA) in the northern Morocco (Table 6). Figs were considered fully ripened when they were easily separated from the twig and when the receptacle turned to reddish-purple coloration. They were picked randomly at different positions around the canopy at height of 160 cm.

### 3.2. Growing Conditions

The cultivars were planted in the same orchard characterized with ferritic soil. During the harvest time, the average air temperature was about 27 °C with important rainfall (26.4 mm) during the last decade of August. Intense solar radiation was observed during the second decade of August and the first decade of September. The ripening process was generally rapid, lasting several days from August to early September, with significant differences among cultivars (Table 6).

### 3.3. Fruit Peel and Pulp Color

The figs’ peels and pulps color were measured using a colorimeter (NH310 colorimeter (Shenzhen 3NH Technology, Shenzhen, China), standardized with white and black calibration. Peel color measurements were obtained from two randomized spots located on opposite sides of the equatorial region of the fruit, while pulp color was measured at two random spots of both half of the fruit. The mean of the two measurements was considered as one replicate. Fifteen replications per sample were considered.

The color was studied in the CIEL*a*b* color space using a Minolta CM-700 (Minolta Camera Co., Osaka, Japan), with illuminant D65, SCI mode and an observer angle of 10°. Low reflectance glass (Minolta CR-A51/1829-752) was placed between the samples and the equipment. The CIEL*a*b* coordinates determined were lightness (L*), redness (a*, coordinate red/green), and yellowness (b*, coordinate yellow-blue) and the psychophysical parameters hab (hue) and Cab∗ (chroma), which were calculated as follows:

$$h_{ab}=arctg\frac{b^{*}}{a^{*}}\;\;\;\;\;C_{ab}^{*}=\sqrt{a^{*2\;}+b^{*2}\;}$$

The present study focused particularly on L*, c* and h° indices, since a* and b* are merely coordinates that indirectly reflect hue and Chroma.

### 3.4. Spectrophotometric Analysis

For each cultivar, figs randomly chosen were manually peeled and each part was frozen at −80 °C for 48 h and then lyophilized (CHRIST ALPHA 1-2 LDplus). Hereto, triplicate lots of fig peels and pulps of each cultivar were grounded to a powder using an IKA A11 Basic Grinder (St. Louis, MO, USA) at room temperature.

#### 3.4.1. Total Phenolics Content (TPC)

Phenolic extraction was performed on the powder of lyophilized peels and pulps as described by Xie and Bolling [42]. TPC was quantified using Folin–Ciocalteu reagent [43] and expressed as mg of gallic acid equivalent (GAE) per 100 g of dry weight (dw).

#### 3.4.2. Total Flavonoids Content (TFC)

TFC was measured using the colorimetric method with aluminum chloride [44] and expressed as (+)-catechin equivalent (CE) per 100 g dw.

#### 3.4.3. Total Anthocyanins Content (TAC)

TAC was measured with the pH differential absorbance method, as described by Cheng and Breen. [45] and calculated using a molar extinction coefficient of 28,800 (cyanidin 3-rutinoside) using the following formula: A = (A520–A700)pH 1.0 − (A520–A700)pH 4.

#### 3.4.4. Total Proanthocyanidins Content (TPAC)

TPAC was determined based on acid hydrolysis and color formation method as reported by Porter et al. [46] and expressed in mg cyanidin equivalent per 100 g dw.

#### 3.4.5. Antioxidant Activity (AA)

The antioxidant activity was assessed as the free radical scavenging activity using two assays DPPH and ABTS in methanolic solution [MeOH/water; 80/20%; v:v; +1% HCl] and the inhibition of lipid peroxidation in linoleic acid system.

The DPPH (radical 2,2-diphenyl-1-picrylhydrazyl) method was performed as described by Brand-Williams et al. [47], while the ABTS [2,2-azinobis-(3-ethylbenzothiazoline- 6-sulphonic acid)] assay carried out as described by Re et al. [48]. The lipid peroxidation inhibition capacity (LPIC) assay was performed as reported by Freire et al. [49]. The mixture’s absorbance was measured at 515, 734 and 470 nm, respectively, using a spectrophotometer (ThermoSpectronic Heγios γ, Cambridge, UK).

For the three assays above, the analyses were performed in triplicate, and the results were expressed as mMol Trolox equivalent/g dw (mMol TE/g dw) using the following formula:

(1) $$\mathrm{mM}\;\mathrm{trolox}\;\mathrm{eq}=\frac{\left(\left({I\%}_{\mathrm{sample}}-b\right)/a\right)\left(\mathrm{mg}/\mathrm{mL}\right)*10^{3}}{c_{\mathrm{sample}}\left(\mathrm{mg}/\mathrm{mL}\right){*M}_{\mathrm{trolox}}\left(g/\mathrm{mol}\right)}$$

where, I(%) represents the percentage inhibition of samples, and a and b correspond, respectively, to the slope and the constant of the linear equation related to the standard curve of each assay.

The half-maximal inhibitory concentration (IC50) was estimated by linear regression using the fitted line as follows:

(2) y=ax+b

(3) $$IC50=\frac{0.5-b}{a}$$

where, *y* represents radical scavenging percentage, and *x* represents samples extracts concentrations.

### 3.5. Polyphenolic Compounds Analysis (PCs)

#### 3.5.1. Extraction Method

Fig samples (1 g) were mixed with 10 mL of methanol: water (80:20, *v/v*). The mixture was sonicated and then macerated for 60 min at 4 °C. The samples were centrifuged for 10 min, 8000 g at 4 °C, and the supernatants were collected, and the pellets were mixed with 10 mL of acetone: water (70:30, *v/v*). The same steps (sonication, maceration and centrifugation) were repeated three times, and the supernatants were combined and evaporated using a rotary evaporator R-205 under reduced pressure, at 40 °C. Five milliliters of methanol were added to the residue, and the mixture was well shaken in a Vortex for 2 min. The samples were filtered through a Sep-Pak (c-18) to remove the sugar content and then were stored at −20 °C until further use.

#### 3.5.2. Determination of PCs

Polyphenolic profiles of both peel and pulp fruits were determined by high-performance liquid chromatography (HPLC) following the methodology described by Genskowsky et al. [50]. A volume of 20 µL of the samples were injected into a Hewlett-Packard HPLC series 1200 instrument equipped with C18 column (Mediterranean sea 18, 25 × 0.4 cm, 5 cm particle size) from Teknokroma, (Barcelona, Spain). Polyphenolic compounds were analyzed, in standard and sample solutions, using a gradient elution at 1 mL/min. The mobile phases were composed by formic acid in water (1:99, *v/v*) as solvent A and acetonitrile as solvent B. The chromatograms were recorded at 280, 320, 360 and 520 nm. Quantitative analysis of phenolic compounds was carried out by reference to authentic standards: gallic acid, (+)-catechin, (−)-epicatechin, chlorogenic acid, quercetin-3-*O*-rutinoside, quercetin-3-*O*-glucoside, luteolin-7-*O*-glucoside, quercetin, apigenin, cyanidin-3,5-diglucoside, cyanidin-3-*O*-rutinoside, pelargonidin-3-*O*-rutinoside (Extrasynthese, Genay, France). Their identification was carried out by comparing UV absorption spectra and retention times of each of them with those of pure standards injected under the same conditions. Each sample was assessed in triplicate, and the results were expressed as µg/g of the dry weight (dw).

### 3.6. Statistical Analysis

Statistical analysis of the data was performed using SPSS v22. Multivariate analysis of variance (MANOVA), with Wilks Lambda used as the test statistic, was performed in the data treatment to test significant differences among cultivars and their fruits’ peels and pulps in addition to their interaction. The differences in the results were estimated with Duncan new multiple range (DMRT) test for pairwise comparison at a level of 5%. A two-dimensional CHA heatmap was applied to the dataset using R software 3.0.2. Prior to this analysis, data were standardized to a comparable scale (µ = 0 and σ = 1). In this presentation of data, the effect size measure is represented by the color intensity. The heatmap groups similar rows and similar columns together, with their similarity represented by a dendrogram. This method is of importance to achieve a better understanding of complex biological systems where one-way direction is assumed [51].

## 4. Conclusions

Understanding the partitioning of phenolic compounds, antioxidant capacity and chromatic coordinates between fig peels and pulps and investigating the relationship between these factors are necessary, since the potential benefits to the consumer health of regular fig intake is to be exploited. In this study, fig samples showed highly significant differences among cultivars and between the fruit parts. The antioxidant potency of these samples was particularly important in peels, where the phenolic compounds are mainly concentrated. All antioxidant test showed a strong correlation with those compounds especially anthocyanins (cyanidine-3.5-diglucoside, cyanidine-3-*O*-rutinoside), that were the predominant compounds in the peel extracts. In pulps samples, (−)-epicatechin and cyanidin-3-*O*-rutinoside were the major compounds. The dark-colored cultivar “INRA 1301” presented the most promising phenolic profile due to its very high levels of almost all detected PCs, especially (−)-epicatechin, quercetin-3-*O*-rutinoside, quercetin-3-*O*-glucoside, cyanidine-3,5-diglucoside and cyanidine-3-*O*-rutinoside. However, it is evident from this study that it is difficult to attribute the antioxidant capacity to one or a specific group of compounds, and that another multifactorial approach is required. Chemometric approaches such as color-coded visualization of the clustered data via dendrograms and heatmaps are of great use to understand the partitioning of studied variables between both cultivars and their fruit parts. The findings herein reported confirm that the figs peels are largely superior to the corresponding pulps, as it relates to phenolic compounds as well as antioxidant potency, endorsing the insistence to further investigate and valorize this unexploited discarded agro-industrial byproduct. They also confirmed the importance of consuming the whole figs as an important habit for the health promoting diet.

## Figures and Tables

**Table 1:** Descriptive analysis and multivariate analysis of variance of all studied variables over figs’ peels and pulps.

Variables	Fruit Part	Mini	Max	Mean	Std. Deviation	ANOVA *p*-Value
Gallic acid *	Peel	0	11.29	0.54	2.24	<0.001
(+)-Catechin *		0	24.06	5.89	5.95	<0.001
(−)-Epicatechin *		2.61	55.44	17.31	12.89	<0.001
Chlorogenic acid *		0	10.67	3.03	2.94	<0.001
Quercetin-3-*O*-rutinoside *		5.3	147.42	58.46	38.66	<0.001
Quercetin-3-*O*-glucoside *		2.52	35.58	11.48	7.76	<0.001
Luteolin-7-*O*-glucoside *		0	18.24	6.75	4.87	<0.001
Quercetin *		0	59.61	4.49	12.48	<0.001
Apigenin *		0	4.91	0.41	1.04	<0.001
Cyanidin-3,5-diglucoside *		0	495.76	48.58	109.91	<0.001
Cyanidin-3-*O*-rutinoside *		0	478.9	46.78	105.29	<0.001
Pelargonidin-3-*O*-rutinoside *		0	12.67	0.67	2.58	<0.001
TPC (mg GAE/100 g dw)		370	3162.86	1368.67	671.01	<0.001
TFC (mg CE/100 g dw)		188.57	2013.57	690.19	371.47	<0.001
TPAC (mg Cyan /100 g dw)		0.2	3.09	0.83	0.83	<0.001
TAC (mg cy-3-r /100 g dw)		4.14	192.5	37.17	41.9	<0.001
DPPH (mMol TE/g dw)		21.23	367.26	156.76	21.53	<0.001
ABTS (mMol TE/g dw)		7.57	563.53	231.52	19.59	<0.001
LPIC (mMol TE/g dw)		139.17	353.11	226.26	10.44	<0.001
L*		19.81	73.51	49.51	15.54	<0.001
c*		0.89	62.76	37.42	16.36	<0.001
h*		-3.41	360.95	78.58	57.02	<0.001
Gallic acid	Pulp	nd	nd	nd	nd	<0.001
(+)-Catechin		0	6.65	1.47	1.4	<0.001
(−)-Epicatechin		1.25	19.06	5.23	4.03	<0.001
Chlorogenic acid		0	4.84	0.77	1.09	<0.001
Quercetin-3-*O*-rutinoside		0	26.85	1.89	5.16	<0.001
Quercetin-3-*O*-glucoside		0	4.05	0.44	0.95	<0.001
Luteolin-7-*O*-glucoside		0	4.5	0.21	0.89	<0.001
Quercetin		nd	nd	nd	nd	<0.001
Apigenin		nd	nd	nd	nd	<0.001
Cyanidin-3,5-diglucoside		0	28.45	5.82	6.68	<0.001
Cyanidin-3-*O*-rutinoside		0.94	34.43	9.01	8.67	<0.001
Pelargonidin-3-*O*-rutinoside		nd	nd	nd	nd	<0.001
TPC		105.71	1255.71	426.38	234.32	<0.001
TFC		13.57	331.43	157.57	79.96	<0.001
TPAC		0.2	1.06	0.37	0.13	<0.001
TAC		2.27	19.44	7.71	4.49	<0.001
DPPH		13.92	151.24	73.99	7.05	<0.001
ABTS		6.59	207.49	76.19	7.35	<0.001
LPIC		42.89	226.88	121.25	7.7	<0.001
L*		12.17	34645	493.3	6.81	0.622
c*		12.35	59.85	28.02	13.64	<0.001
h*		4.87	74.8	32.33	16.59	<0.001
**Effect**	**Wilks Lambda’s value**	**F**	**Hypothesis df**	**Error df**	**Sig.**	
Variety	0	477.23	560	1376.367	0	
Fruit part	0	496,075.72	20	79	0	
Variety * Fruit part	0	464.37	440	1242.807	0	

* expressed as µg/g of dry weight; nd: not detected; df: degree of liberty; F: refers to Fisher statistic; Sig.: signification; Cyan: cyanidin; cy-3-r: Cyanidin-3-rutinoside.TPC: total phenolics content; TFC: total flavonoids content; TAC: total anthocyanins content; TPAC: Total proanthocyanidins content; DPPH: 2,2-diphenyl-1-picrylhydrazyl; ABTS: 2,2-azinobis-(3-ethylbenzothiazoline-6-sulphonic acid; LPIC: lipid peroxidation inhibition capacity

**Table 2:** Total phenols, flavonoids, anthocyanins, proanthocyanidins, antioxidant activity and chromatic coordinates of fig peels.

Cultivars	TPC	TFC	TPAC	TAC	DPPH	ABTS	LPIC	IC50 (DPPH)	IC50 (ABTS)	IC50 (LPIC)	L*	c*	h°
Bioudie	1346.19	602.86	0.90	10.82	332.96	452.52	154.84	172.38	292.70	114.83	55.46	45.97	91.43
Breba Blanca	1093.81	590.95	0.43	14.27	100.19	192.06	278.94	125.46	185.47	83.56	59.81	47.67	85.01
Breval Blanca	796.19	669.52	0.51	15.78	254.75	364.80	231.87	306.04	285.62	24.45	62.62	54.83	90.86
Chaari	696.19	388.57	0.39	9.93	299.74	527.25	169.11	129.47	112.22	51.81	44.85	39.14	90.87
Chetoui	1100.95	708.81	2.20	18.13	329.04	336.61	160.55	27.42	119.33	124.80	65.56	50.4	96.1
Cuello Dama Blanco	1391.43	1177.86	0.33	34.88	333.99	491.90	155.56	80.47	163.46	86.35	63.32	51.12	92.73
El Quoti Lbied	1241.43	493.33	0.50	11.65	68.83	175.95	255.40	156.99	154.30	99.83	48.8	37.29	95.72
Fassi	2020.00	935.00	0.58	47.90	332.13	493.69	232.58	0.28	97.35	216.92	31.78	10.28	215.93
Ghoudan	927.14	602.86	0.46	19.16	133.21	210.85	289.63	76.47	225.29	81.13	28.13	28.96	42.36
Herida	389.05	214.76	0.33	21.50	56.86	77.49	240.43	234.03	230.18	125.68	51.25	44.53	90.85
INRA 1302	1627.14	807.62	1.97	53.00	37.87	77.49	191.22	3.97	56.49	76.19	31.36	18.39	18.33
INRA 1305	912.86	417.14	1.04	22.33	40.76	157.60	216.89	92.67	114.38	136.79	36.42	19.02	36.25
INRA 2105	2070.00	1282.62	1.67	51.97	79.97	137.01	251.84	3.88	73.70	101.26	34.61	13.85	19.96
INRA 2201	1865.24	689.76	1.13	126.41	54.38	147.31	295.34	109.17	68.01	263.49	36.79	24.53	25.41
INRA 2304	2396.19	385.00	0.76	63.75	16.62	91.81	238.29	2.12	21.84	124.04	24.52	38.88	23.35
INRA1301	2860.48	1944.52	2.59	192.23	152.40	190.72	198.35	19.85	40.58	124.78	25.72	22.09	20.99
Kadata	1208.10	492.14	0.54	17.16	29.41	126.27	301.76	201.52	174.22	98.09	59.62	48.67	98.26
Nabout	810.48	699.29	1.21	82.29	121.45	459.23	239.00	16.98	177.38	132.85	52.86	55.04	89.07
Noukali	822.38	677.86	0.50	21.23	128.26	212.64	203.34	25.04	202.71	55.03	28.7	41.67	53.68
Ouarraksi	1384.29	763.57	0.48	4.82	218.02	293.65	278.94	161.00	297.22	88.32	59.26	38.39	103.53
Palmeras	2855.71	1070.71	0.33	18.06	29.21	157.15	199.06	106.34	179.49	87.90	65.15	36.95	93.99
Sarilop	1155.71	263.57	0.38	17.16	21.78	12.82	278.22	175.77	200.03	78.28	59.21	48.26	90.94
Snowden	1700.95	598.10	0.42	15.58	69.45	440.43	248.98	123.15	190.12	80.01	72.62	54.57	94.84
Trojana	415.24	317.14	0.56	11.10	5.27	12.01	189.79	170.40	495.99	295.07	73.16	50.95	90.38
White Adriatic	1129.52	461.19	0.44	28.05	22.66	0.07	156.27	162.94	299.49	103.07	59.7	37.58	99.14

TPC: total phenolics content (mg GAE/100 g dw); TFC: total flavonoids content (mg CE/100 g dw); TPAC; total proanthocyanidins content (mg cyanidin equivalent/100 g dw); TAC: total anthocyanins content (mg cyanidin-3-rutinoside eq/100 dw); DPPH, ABTS and LPIC were expressed in mMol trolox/g dw; IC50 was expressed in µg/mL.

**Table 3:** Total phenols, flavonoids, anthocyanins, proanthocyanidins, antioxidant activity and chromatic coordinates of fig pulps.

Cultivar	TPC	TFC	TPAC	TAC	DPPH	ABTS	LPIC	IC50 (DPPH)	IC50 (ABTS)	IC50 (LPIC)	L*	c*	h°
Bioudie	339.05	95.71	0.40	3.58	22.21	24.37	40.48	275.08	418.35	152.16	20.67	44.04	7.83
Breba Blanca	255.71	170.71	0.36	5.86	19.43	24.32	35.86	206.83	226.64	231.46	34.91	17.05	36.51
Breval Blanca	731.90	257.62	0.45	6.75	35.01	16.42	23.51	163.29	455.25	169.11	19.05	55.05	16.46
Chaari	284.29	250.48	0.26	3.03	25.41	23.44	24.85	202.38	220.80	241.85	34.10	29.50	32.35
Chetoui	491.43	257.62	0.64	8.27	39.50	25.03	38.99	168.57	204.48	231.55	25.18	15.33	27.07
Cuello Dama Dlanco	439.05	93.33	0.25	3.45	33.75	40.83	21.13	199.52	221.21	238.04	19.95	46.79	6.52
El Quoti Lbied	422.38	162.38	0.41	3.24	32.69	33.51	21.43	139.85	198.06	201.63	44.51	16.53	42.33
Fassi	234.29	93.33	0.38	11.37	27.66	22.73	31.40	245.24	201.32	130.18	18.62	44.07	36.60
Ghoudan	1186.67	271.90	0.29	12.48	33.60	33.95	32.59	201.06	241.50	85.47	33.24	14.72	54.88
Herida	174.76	107.62	0.29	7.72	18.70	17.67	35.86	151.47	233.68	148.96	34.18	15.14	62.97
INRA 1302	520.00	250.48	0.47	7.31	34.06	35.82	26.79	167.29	316.70	392.39	35.10	22.29	36.35
INRA 1305	262.86	131.43	0.22	16.89	15.35	14.43	43.45	269.32	665.58	138.37	40.04	17.22	55.95
INRA 2105	753.33	160.00	0.56	9.58	38.78	32.85	33.33	117.46	138.88	164.76	34.31	17.98	38.18
INRA 2201	374.76	106.43	0.43	3.24	25.75	33.20	35.12	160.10	186.17	145.74	45.91	18.83	42.91
INRA 2304	465.24	229.05	0.31	5.17	30.21	23.26	24.55	273.64	299.00	138.24	41.24	27.78	40.09
INRA1301	398.57	186.19	0.41	14.06	31.43	22.99	35.42	124.58	309.90	149.39	36.55	19.90	39.46
Kadata	331.90	146.90	0.43	10.68	29.18	26.01	24.55	152.18	225.52	187.61	33.31	14.83	33.58
Nabut	662.86	285.00	0.42	3.10	25.22	31.69	28.27	202.18	292.24	241.83	27.72	40.87	24.90
Noukali	329.52	21.90	0.30	5.03	30.51	28.81	23.96	290.33	242.72	212.67	20.45	46.30	23.42
Ouarraksi	255.71	137.38	0.55	3.58	26.48	22.50	18.01	173.75	288.42	306.69	37.63	31.32	35.36
Palmeras	450.95	199.29	0.22	11.85	32.38	29.69	30.36	187.54	138.48	185.15	19.22	49.20	13.68
Sarilop	408.10	110.00	0.26	5.44	24.23	20.86	16.07	308.37	353.84	300.35	41.90	22.04	57.91
Snowden	274.76	20.71	0.39	10.34	27.01	35.86	26.49	152.55	359.54	138.24	36.62	15.71	44.56
Trojana	205.71	133.81	0.31	3.86	19.54	15.85	28.57	563.91	892.67	231.46	24.06	34.28	15.21
White Adriatic	405.71	60.00	0.35	16.82	42.63	24.01	31.40	166.52	415.02	125.67	38.22	20.90	46.45

TPC: total phenolics content (mg GAE/100 g dw); TFC: total flavonoids content (mg CE/100 g dw); TPAC; total proanthocyanidins content (mg cyanidin equivalent/100 g dw); TAC: total anthocyanins content (mg cyanidin-3-rutinoside eq/100 dw); DPPH, ABTS and LPIC were expressed in mMol trolox/g dw; IC50 was expressed in µg/mL.

**Table 4:** Contents of individual phenolic compounds (µg/g dw) among cultivars figs peels.

Cultivars	Gallic Acid	(+)-Catechin	(−)-Epicatechin	Chlorogenic Acid	Quercetin-3-*O*-rutinoside	Quercetin-3-*O*-Glucoside	Luteolin-7-*O*-Glucoside	Quercetin	Apigenin	Cyanidin-3,5-Diglucoside	Cyanidin-3-*O*-Rutinoside	Pelargonidin-3-*O*-Rutinoside	Total (µg/g)
Biondie	-	3.38	14.05	3.34	55.39	11.66	7.19	0.86	-			-	95.86
Breba Blanca	-	1.74	6.37	0.56	10.98	6.16	-	-	-	0.86	1.06	-	27.74
Breval Blanca	-	5.14	19.01	1.66	65.05	7.29	12.43	-	-	0.76	0.84	-	112.18
Chaari	-	1.30	9.84	0.59	44.18	10.07	5.83	-	-	3.76	6.31	-	81.88
Chetoui	11.25	2.86	10.21	2.93	125.68	18.66	8.96	-	-	0.86	1.08	-	182.49
Cuello Dama Blanco	-	16.54	42.35	8.77	46.49	5.32	17.91	59.52	4.84	-	-	-	201.73
El Quoti Lbied	-	2.81	16.24	0.49	28.94	6.92	-	1.01	1.13	7.03	-	-	64.57
Fassi	-	7.76	19.01	4.12	147.33	21.06	15.21	1.00	-	81.08	83.91	-	380.49
Ghoudan	-	2.35	16.67	1.16	85.87	21.91	11.86	1.40	-	21.06	20.17	-	182.46
Herida	-	0.99	5.12	0.46	17.88	3.39	2.58		-			-	30.42
INRA 1302	-	4.02	12.23	4.28	53.78	10.34	5.69	4.45	-	97.69	100.32	-	292.81
INRA 1305	-	3.82	18.96	8.71	70.98	14.67		1.17		21.14	21.61	-	161.05
INRA 2105	-	23.87	13.18	0.67	38.35	8.63	3.82	5.52	1.70	43.03	40.55	-	179.31
INRA 2201	-	4.67	24.83	5.49	54.77	13.46	5.82	1.20		275.20	246.86	4.16	632.31
INRA 2304	-	7.45	11.07	0.86	64.49	14.19	7.26	3.87		130.12	131.23	-	370.54
INRA1301	-	14.36	54.66	7.81	141.08	35.48	11.50	2.17		494.08	478.66	12.56	1239.80
Kadata	-	0.92	6.21	1.40	20.23	2.56	3.34	0.91	1.11			-	36.69
Nabout	2.22	7.01	9.86	0.90	33.78	6.03	6.71	1.46	-	1.04	1.15	-	70.15
Noukali	-	6.28	31.98	8.39	112.91	21.47	1.86	-	-	35.89	34.88	-	253.66
Ouarraksi	-	3.19	12.42	3.71	92.49	11.22	8.61	-	-	0.83	0.94	-	133.41
Palmeras	-	18.25	44.22	4.48	23.15	18.31	15.03	-	1.40	-	-	-	151.77
Sarilop	-	3.60	14.91	2.30	41.88	4.64	4.30	-	-	-	-	-	71.63
Snowden	-	2.83	11.52	2.24	49.46	6.86	5.33	-	-	-	-	-	78.23
Trojana	-		2.67		16.47	3.25	2.39	-	-	-	-	-	24.78
White Adriatic	-	2.24	5.09	0.57	19.79	3.53	5.17	0.91	-	-	-	-	37.30

-: Not detected.

**Table 5:** Contents of individual phenolic compounds (µg/g dw) among cultivars’ fig pulps.

Cultivars (Pulp)	(+)-Catechin	(−)-Epicatechin	Chlorogenic Acid	Quercetine-3-*O*-Rutinoside	Quercetine-3-*O*-Glucoside	Luteoline-7-*O*-Glucoside	Cyanidine-3.5-Diglucoside	Cyanidine-3-*O*-Rutinoside	Total (µg/g)
Biondie	-	2.15	-	-	-	-	0.82	1.31	1.43
Breba Blanca	1.12	3.38	0.46	-	-	-	3.86	1.85	2.13
Breval Blanca	1.71	4.79	0.33	0.92	-	-	5.53	8.05	3.56
Chaari	1.43	4.09	-	1.23	-	-	2.59	3.8	2.63
Chetoui	1	7.99	1.3	26.8	4.03	0.75 ± 0.35	6.87	11.87	8.55
Cuello Dama Blanca	-	3.85	3.14	1.23	0.98	-	4.74	11.05	4.17
El Quoti Lbied	-	1.75	0.38	-	-	-	3.91	7.93	3.49
Fassi	2.63	9.08	0.68	1.23	-	-	11.93	16.94	7.08
Ghoudan	1.92	2.47	0.31	1.51	1.31	-	2.41	5.42	2.19
Herida	0.67	2.03	-	-	-	-	0.9	1.04	1.16
INRA 1302	2.96	9.91	1.22	1.06	-	-	14.74	20.37	8.38
INRA 1305	2.11	7.83	1.04	-	-	-	-	1.52	3.13
INRA 2105	6.63	19.05	4.83	2.21	1.45	-	20.38	28.78	11.90
INRA 2201	1.34	1.75	0.4	-	-	-	4.73	10.44	3.73
INRA 2304	-	1.27	-	0.81	-	-	2.83	5.12	2.51
INRA 1301	0.73	1.3	-	-	-	-	0.83	1.39	1.06
Kadota	1.2	8.37	0.44	1.02	-	-	8.98	16.6	6.10
Nabout	1.5	2.52	0.36	1.03	1.05	-	1.11	3.03	1.51
Noukali	1.67	4.28	0.33	1.48	-	-	6.15	11.1	4.17
Ouarraksi	1.39	1.65	0.47	1.42	-	-	4.11	9.68	3.12
Palmeras	3.06	7.23	2.07	1.92	2.19	4.47 ± 0.04	6.88	8.81	4.59
Sarilop	1.82	10.88	0.49	0.89	-	-	0.93	2.25	2.88
Snowden	-	5.08	0.37	-	-	-	0.89	1.57	1.98
Trojana	-	4.15	-	1.2	-	-	0.84	0.96	1.79
White Adriatic	1.83	3.91	0.58	1.22	-	-	28.43	34.42	11.73

-: Not detected.

**Table 6:** Cultivars geographical origins, harvest time and monthly meteorological data from August to early September 2018 in Northern Morocco, Meknes (Ain-Taoujdate experimental station—INRA).

	Cultivars	Geographical Origin	August						September	
			(1–5)	(6–10)	(11–15)	(16–20)	(21–25)	(26–30)	(31–4)	(5–9)
Local	El Quoti Lbied	Morocco								
	Nabout									
	Fassi									
	Noukali									
	Ghoudan									
	Chetoui									
	Bioudie									
	Chaari									
	Ournaksi									
	INRA 1305									
	INRA 2105									
	INRA 1302									
	INRA 2201									
	INRA 2304									
	INRA 1301									
Introduced	Snowden	USA								
	White Adriatic	Italy								
	Kadota	Italy								
	Triana	Italy								
	Cuello Dama Blanca	Spain								
	Breval Blanca	Spain								
	Palmeras	Spain								
	Herida	Spain								
	Breba Blanca	Spain								
Total rainfall (mm)			0	0	0	0	0	26.4	0	0
Average temperature (°C)			25.84	28.5	27.56	29.24	29.44	23.64	25.6	25.42
Average solar radiation (W/m²)			169.29	208.74	243.83	238.28	185.35	123.5	270.21	271.38
Soil type			Sandy clay loam with an average organic matter of 1% [0–30 cm soil layer]							
Soil pH			7.2							

Climatic data collected from meteorological station installed next to the orchard. Texture organic matter was assessed over a composite sample using Walkley and Black [52] and Robinson methods, respectively. The green color describes the maturity period of each fig trees herein investigated in term of number of days (each column represents five days).

## Data Availability

Not Avaliability.

## Supplementary Materials

The following are available online, Figure S1: Heatmap correlation between all studied variables in fig pulp samples. Figure S2: Heatmap correlation between all studied variables in fig peel samples. Blue color refers to the positive correlations, while red one indicates low correlations between variables. For both, the low color intensity means the lower value and vice versa.
