# Supplementary material for: Survey of Phenolic Acids, Flavonoids and In Vitro Antioxidant Potency Between Fig Peels and Pulps: Chemical and Chemometric Approach
Source: Molecules. 2021 Apr 28;26(9):2574. doi: 10.3390/molecules26092574 (PMC8124568; doi:10.3390/molecules26092574)
Supplement: Supplementary file 1 [file molecules-26-02574-s001.zip › molecules-1020853-supplementary.pdf]

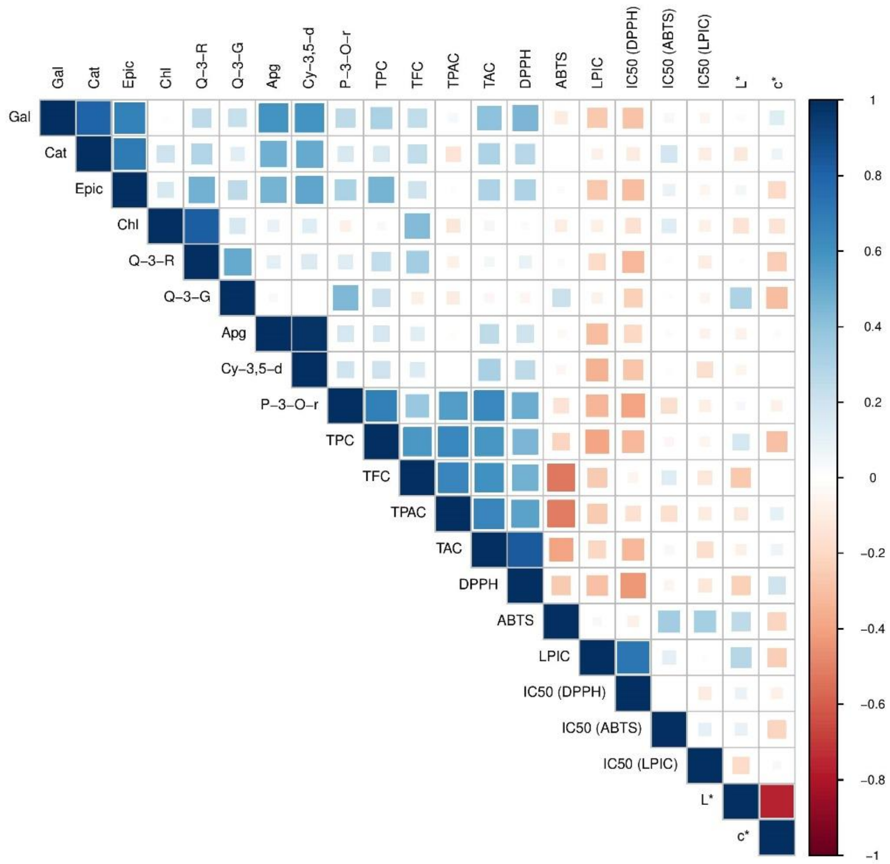

Figure S1. Heatmap correlation between all studied variables in fig pulp samples. Blue color refers to the positive correlations, while red one indicates low correlations between variables. For both, the low color intensity means the lower value and vice versa. Chr.A: chlorogenic acid; Q.3.O.r: quercetin-3-O-rutinoside; Q.3.O.g: quercetin-3-O-glucoside; Lu.7.O.g: luteolin-7-O-glucoside; Quercetin: quercetin; Apigenin: apigenin; Cya.3,5.d: cyanidin-3,5-diglucoside; Cya.3.O.r: cyanidin-3-O-rutinoside; Pel.3.O.r: pelargonidin-3-O-rutinoside.”.

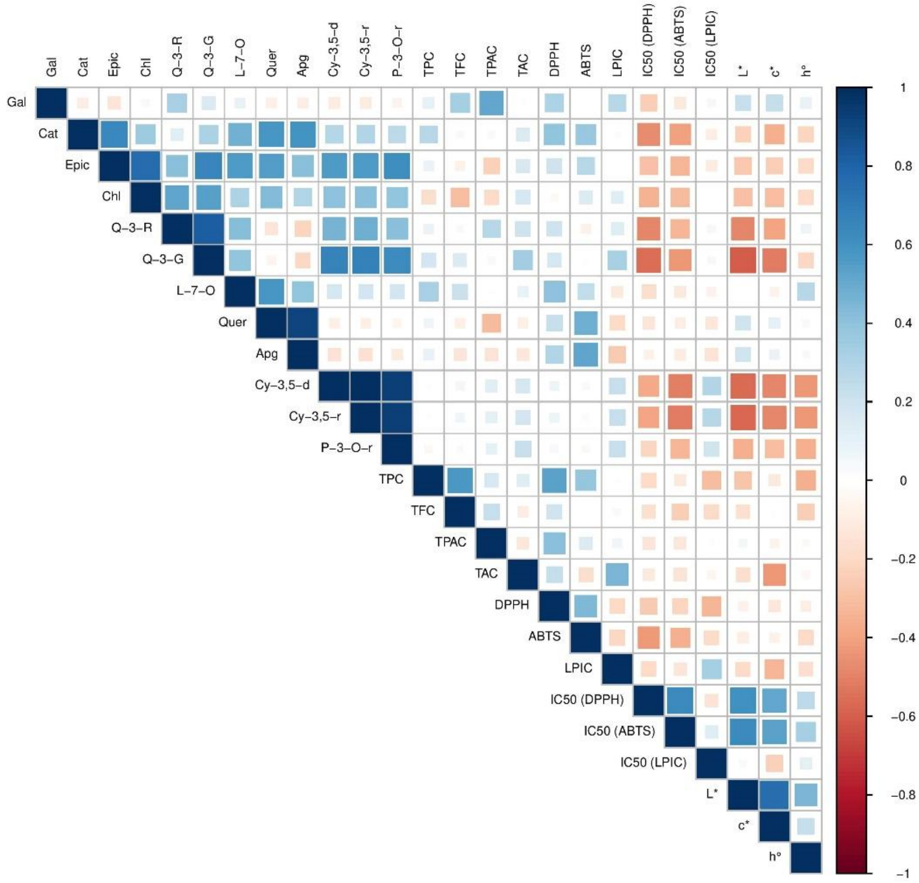

Figure S2. Heatmap correlation between all studied variables in fig peel samples. Blue color refers to the positive correlations, while red one indicates low correlations between variables. For both, the low color intensity means the lower value and vice versa. Chr.A: chlorogenic acid; Q.3.O.r: quercetin-3-O-rutinoside; Q.3.O.g: quercetin-3-O-glucoside; Lu.7.O.g: luteolin-7-O-glucoside; Quercetin: quercetin; Apigenin: apigenin; Cya.3,5.d: cyanidin-3,5-diglucoside; Cya.3.O.r: cyanidin-3-O-rutinoside; Pel.3.O.r: pelargonidin-3-O-rutinoside.”.
